# Biochar-bacteria-plant combined potential for remediation of oil-contaminated soil

**DOI:** 10.3389/fmicb.2024.1343366

**Published:** 2024-05-21

**Authors:** Xin Fang, Mei Zhang, Pufan Zheng, Haomin Wang, Kefan Wang, Juan Lv, Fuchen Shi

**Affiliations:** ^1^College of Life Sciences, Nankai University, Tianjin, China; ^2^Key Laboratory of Storage and Preservation of Agricultural Products, Ministry of Agriculture and Rural Affairs, Tianjin Key Laboratory of Postharvest Physiology and Storage and Preservation of Agricultural Products, Institute of Agricultural Products Preservation and Processing Technology, Tianjin Academy of Agricultural Sciences (National Research Center of Agricultural Products Preservation Engineering and Technology (Tianjin)), Tianjin, China; ^3^School of Environmental Science and Engineering, Tiangong University, Tianjin, China

**Keywords:** bioremediation, oil-contaminated soil, microbial community, total petroleum hydrocarbons, rhizosphere

## Abstract

Oil pollution is a common type of soil organic pollution that is harmful to the ecosystem. Bioremediation, particularly microbe-assisted phytoremediation of oil-contaminated soil, has become a research hotspot in recent years. In order to explore more appropriate bioremediation strategies for soil oil contamination and the mechanism of remediation, we compared the remediation effects of three plants when applied in combination with a microbial agent and biochar. The combined remediation approach of *Tagetes erecta*, microbial agent, and biochar exhibited the best plant growth and the highest total petroleum hydrocarbons degradation efficiency (76.60%). In addition, all of the remediation methods provided varying degrees of restoration of carbon and nitrogen contents of soils. High-throughput sequencing found that microbial community diversity and richness were enhanced in most restored soils. Some soil microorganisms associated with oil degradation and plant growth promotion such as *Cavicella*, *C1_B045*, *Sphingomonas*, *MND1*, *Bacillus* and *Ramlibacter* were identified in this study, among which *Bacillus* was the major component in the microbial agent. *Bacillus* was positively correlated with all soil remediation indicators tested and was substantially enriched in the rhizosphere of *T. erecta*. Functional gene prediction of the soil bacterial community based on the KEGG database revealed that pathways of carbohydrate metabolism and amino acid metabolism were up-regulated during remediation of oil-contaminated soils. This study provides a potential method for efficient remediation of oil-contaminated soils and thoroughly examines the biochar–bacteria–plant combined remediation mechanisms of oil-contaminated soil, as well as the combined effects from the perspective of soil bacterial communities.

## Introduction

With the rapid progress of society and the development of scientific technology, environmental pollution is receiving increasing attention. Oil pollution caused by leakage during oil transportation and industrial production processes such as oil extraction and oil processing is a hot topic of research and concern. Crude oil contains various carcinogenic, teratogenic, and mutagenic substances, including petroleum alkanes, cycloalkanes, and aromatic hydrocarbons, which can cause serious damage to the environment (Goveas et al., 2020; Sarfo et al., 2023). Spilled oil can swiftly seep into soil pores and be absorbed by soil grains, which markedly affects the carbon, nitrogen, and phosphorus contents, pH, aeration, and other physicochemical properties of the soil (Rhykerd et al., 1999; Li et al., 2015). Moreover, oil pollution has a significant impact on the establishment and succession of native plant communities, particularly in habitats with extreme environmental conditions such as salty soils (Liu et al., 2021; Novakovskiy et al., 2021). Oil pollution may also endanger the health of animals and even humans through the food chain (Li H. S. et al., 2022). Therefore, how to remediate oil pollution is a major focus of contemporary research.

Bioremediation is currently an important approach to remove oil pollution. Bioremediation measures commonly used to rectify oil pollution mainly include phytoremediation and microbial remediation. Phytoremediation utilizes a series of processes during growth such as biodegradation, extraction, and phytovolatilization of petroleum by plants, as well as the enrichment of soil microorganisms in rhizospheres, to remediate oil contamination in soil (Yeh et al., 2015; Chen et al., 2017; Fahid et al., 2020). Phytoremediation is one of the most economical and environmentally friendly approaches for bioremediation. However, this approach has a long remediation cycle owing to its natural growth limitations and is less effective in remediating high concentrations of pollution (Iffis et al., 2017; He et al., 2022). Microorganisms are promising degraders of organic pollutants (Bhatt et al., 2020), and microbial remediation is a technique that utilizes microorganisms to mitigate, degrade, or reduce harmful contaminants to innocuous compounds (Varjani, 2017). The strategies for microbial remediation widely used to remediate oil pollution mainly include bioaugmentation, biostimulation, and other techniques such as remediation by enhanced natural attenuation. Bioaugmentation involves replenishing and remediating the microbial community by adding microorganisms capable of degrading oil pollutants; and biostimulation refers to stimulating the growth of oil-degrading microorganisms primarily through the addition of nutrients or other substrates necessary for microbial growth (Agarry et al., 2010; Nwankwegu and Onwosi, 2017; Zeneli et al., 2019). The degradation effect of different types of oil pollution by different microorganisms varies significantly, and environmental factors like moisture, temperature, and nutrition also affect the degradation ability of microorganisms (Varjani, 2017; Xu et al., 2018; Okoh et al., 2020).

Consequently, the combined plant–microbe remediation system has progressively gained in popularity and developed into a hotspot in the field of bioremediation research (Zhang et al., 2014; Cheng et al., 2019). Currently, numerous studies are being conducted to find combinations of plants and microorganisms that can efficiently degrade oil pollution and have better adaptability to the natural environment at the same time (Zhang et al., 2021; Zhao et al., 2021; Mfarrej et al., 2023). In a combined plant–microbe remediation system, the rhizosphere is the main bioactive region. According to Gerhardt et al. (2009), rhizoremediation is a specific process in which plants and their associated rhizosphere microbes cooperate to remediate soil contamination. Root exudates can also be crucial in this process. In the hypothesis proposed by Heinonsalo et al. (2000), root and mycorrhizosphere development in lignin-rich forest humus and petroleum hydrocarbon-contaminated soils positively affect the way bacteria use carbon sources, and are crucial for bacterial community succession and degradation of petroleum pollution. In the co–application of microorganisms and plants for the remediation of soil oil contamination, microorganisms can improve the tolerance of plants to oil pollution, whereas plants can reshape the structure of soil microbial communities, which can enhance the effect of oil degradation and provide the possibility for bioremediation in more complex environments (Jimoh et al., 2022; Wang et al., 2022).

At present, the combined plant–microbe remediation system has many challenges that deserve further exploration. For instance, some researchers have examined the effect of root exudates of plants on the activity and growth of microorganisms, with Chetverikov et al. (2021) noting that bacterial activity could also influence the secretion of root exudates. Therefore, the selection of plant and bacterial species for bioremediation requires consideration of such information. Besides, in practical applications of the combined plant–microbe remediation system, suitable soil amendments may still be required. Biochar, which is produced by pyrolyzing natural biomass materials like plant and animal residues, is a soil amendment that is frequently used in pollution remediation. Biochar increases soil fertility and lessens the stress that oil pollution places on plants (Kim et al., 2018; Ren et al., 2020). In the combined application of biochar and microorganisms, biochar can provide a carbon source for microorganisms and can also adsorb pollutants to facilitate the contact between the microorganisms and the pollutants (Zhu et al., 2017). Furthermore, adding biochar is more likely to produce dominant bacterial communities for degrading pollutants in contaminated soils, which has a good application prospect in the adsorption and degradation of soil pollution (Tomczyk et al., 2020; Dike et al., 2022). Based on the above information, the objectives of this study were: to select the more appropriate plant to work with microorganisms for efficient remediation of soil oil contamination, to explore the mechanism underlying the interaction between plant and microorganisms, and to determine whether the addition of biochar could enhance the remediation effect of combined plant–microbe remediation.

## Materials and methods

### Experiment design and sample collection

Experiments were performed in Nankai University Horticultural Experimental Station (39°6′ N, 117°10′ E), Tianjin, China. Three plants were selected for the pot experiment for bioremediation of oil contamination: *Hibiscus moscheutos*, *Mirabilis jalapa*, and *Tagetes erecta*. These three plants are widely acknowledged as having some pollution remediation capacity and are also ornamental. Hairuike compound microbial agent produced by Yangzhou Hairuike Limited Company was selected as the source of microorganisms to degrade oil pollution, and was a lyophilized formulation mostly consisting of *Bacillus*, *Micrococcus*, enzymes, and nutrients. Biochar produced from corn stover was also utilized in the study. The experimental treatments of the three plant species, microbial agent, and biochar designed to remediate oil-contaminated soil by individual and combined applications are shown in Table 1.

**Table 1:** The design of experimental treatments.

	Control	*Hibiscus moscheutos*	*Mirabilis jalapa*	*Tagetes erecta*
Uncontaminated soil	C	HMC	MJC	TEC
Oil-contaminated soil	P1	HMP1	MJP1	TEP1
Oil-contaminated soil containing microbial agent	P2	HMP2	MJP2	TEP2
Oil-contaminated soil containing biochar	P3	HMP3	MJP3	TEP3
Oil-contaminated soil containing microbial agent and biochar	P4	HMP4	MJP4	TEP4

The abbreviations in the table represent the names of the treatments that combined the application of the remediation methods (plants, microbial agent, and biochar) shown in the table. No plants were planted in the control treatments.

Each treatment was designed with three pot replicates, with each pot containing 1.5 kg of culture soil. Culture soil was provided by the Beijing Jiahui Landscaping and Flower Company (Beijing, China), constituted of garden soil, river sand, decompressed cow dung and peat in a ratio of 4:2:1:1(volume ratio). Culture soil was sterilized by autoclaving before use. Before planting, 5.5 g of crude oil was added to each pot and soil samples were taken to determine the total petroleum hydrocarbons (TPH) content to ensure that the soil TPH content was around 3.5 g/kg. The microbial agent was added at 5% of the dry weight of soil per pot, before which the microbial agent was diluted 1:10 with distilled water and was incubated with aeration and 10 mM glucose addition for 8 h. Biochar was added at 4% of the dry weight of soil per pot. Healthy seedlings of similar growth were transplanted into pots (three seedlings per pot) in June 2022. The experiment was conducted from June to September 2022 in Nankai University Horticultural Experimental Station under natural temperature and sunlight, with an average high temperature of 30.25°C, an average low temperature of 22.25°C, and an average sunshine duration of 13.63 h. And watered each pot with equal amounts of water every day to maintain the soil moisture at around 50%. Plant growth parameters and physiological indicators were measured, and the plants were harvested after 90 d of growth. Soil samples for microbial analysis were collected at the same time as the plants were harvested. The entire plant root system was dug out and patted until all the loose soil clods fell off. Soil attached to the root surface was then collected as rhizosphere soils using sterile brushes. The non-rhizosphere soil was taken at a depth of 5–10 cm of the pot. Thereafter the soil samples were kept at −80°C for microbial analysis. Remaining soil in the pot was air-dried and sieved through a 2-mm mesh pending subsequent physical and chemical qualities determination.

### Detection of soil physicochemical indicators and plant growth parameters

Total petroleum hydrocarbons (TPH) of the oil-contaminated soils were determined by ultrasonic extraction of soil samples with 30 mL of dichloromethane for 15 min, centrifugation at 4000 rpm for 10 min, and evaporation of the supernatant to dryness at 54°C for gravimetric determination. Total carbon (TC), total nitrogen (TN) and carbon-to-nitrogen ratio (C/N) were detected by the Vario MICRO cube element analyzer (Elementar, Germany). The height of the plants was measured on the day 90 after planting and the average of three plants in each pot was taken. All plants were then harvested and dried to measure biomass. Each indicator was determined in triplicate.

### Bioinformatics analysis

Soil samples kept at −80°C were sent to Biomarker Technology Co., Ltd. (Beijing, China) for DNA isolation and sequencing. DNA in the soil samples was extracted with a TGuide S96 Magnetic Soil Genomic DNA Kit (Tiangen Biotech (Beijing) Co., Ltd.) according to the manufacturer’s instructions. The V3–V4 region of the 16S rRNA gene was amplified by primers 338F (5′-ACTCCTACGGGAGGCA GCA-3′) and 806R (5′-GGACTACHVGGGTWTCTAAT-3′) with barcodes. The PCR system contained 5–50 ng of DNA template, 0.3 μg of aforementioned primers (10 μM each), 5 μL of KOD FX Neo Buffer, 2 μL of dNTPs (2 mM each), 0.2 μL of KOD FX Neo, and supplemental ddH_2_O to 10 mL. The PCR cycle comprised an initial denaturation at 95°C for 5 min, followed by 25 cycles of denaturation at 95°C for 30 s, annealing at 50°C for 30 s, and extension at 72°C for 40 s, then a final extension at 72°C for 7 min. Agencourt AMPure XP Beads (Beckman Coulter, Indianapolis, IN, USA) were used to purify all the PCR amplicons and a Qubit dsDNA HS Assay Kit and Qubit 4.0 Fluorometer (Invitrogen, Thermo Fisher Scientific, OR, USA) was used to quantify the DNA. The constructed library was then sequenced using an Illumina NovaSeq6000 system (Illumina, Santiago, CA, USA) at Biomarker Technology Co., Ltd. (Beijing, China). Sequences with similarity >97% were clustered into the same operational taxonomic unit (OTU) using USEARCH (v10.0) and OTUs with abundance <0.005% were filtered and removed. Based on the SILVA database (version 132), the remaining OTUs were classified and annotated using Naive Bayes classifier in QIIME2 with a confidence threshold of 70%.

### Statistical analysis

Microsoft Excel 2021 was used for data collation and SPSS 22.0 was used for data processing and analysis. The significance of differences was tested using analysis of variance (ANOVA) and Duncan’s tests. Multivariate variance analysis was also performed in SPSS 22.0. Origin 2023 was used for graphing of TPH degradation efficiency, plant biomass, plant height, relative abundance of soil bacteria, and correlation analysis. Alpha diversity and beta diversity were calculated and displayed by the QIIME2 and R software, respectively. Linear discriminant analysis (LDA) effect size (LefSe) analysis was performed by LefSe tools on the platform BMKCloud and a logarithmic LDA score of 4.0 was set as the threshold for discriminative features. A functional gene prediction heatmap was also analyzed on the BMKCloud platform and graphed by Origin 2023.

## Results

### TPH degradation efficiency

**Figure 1:**
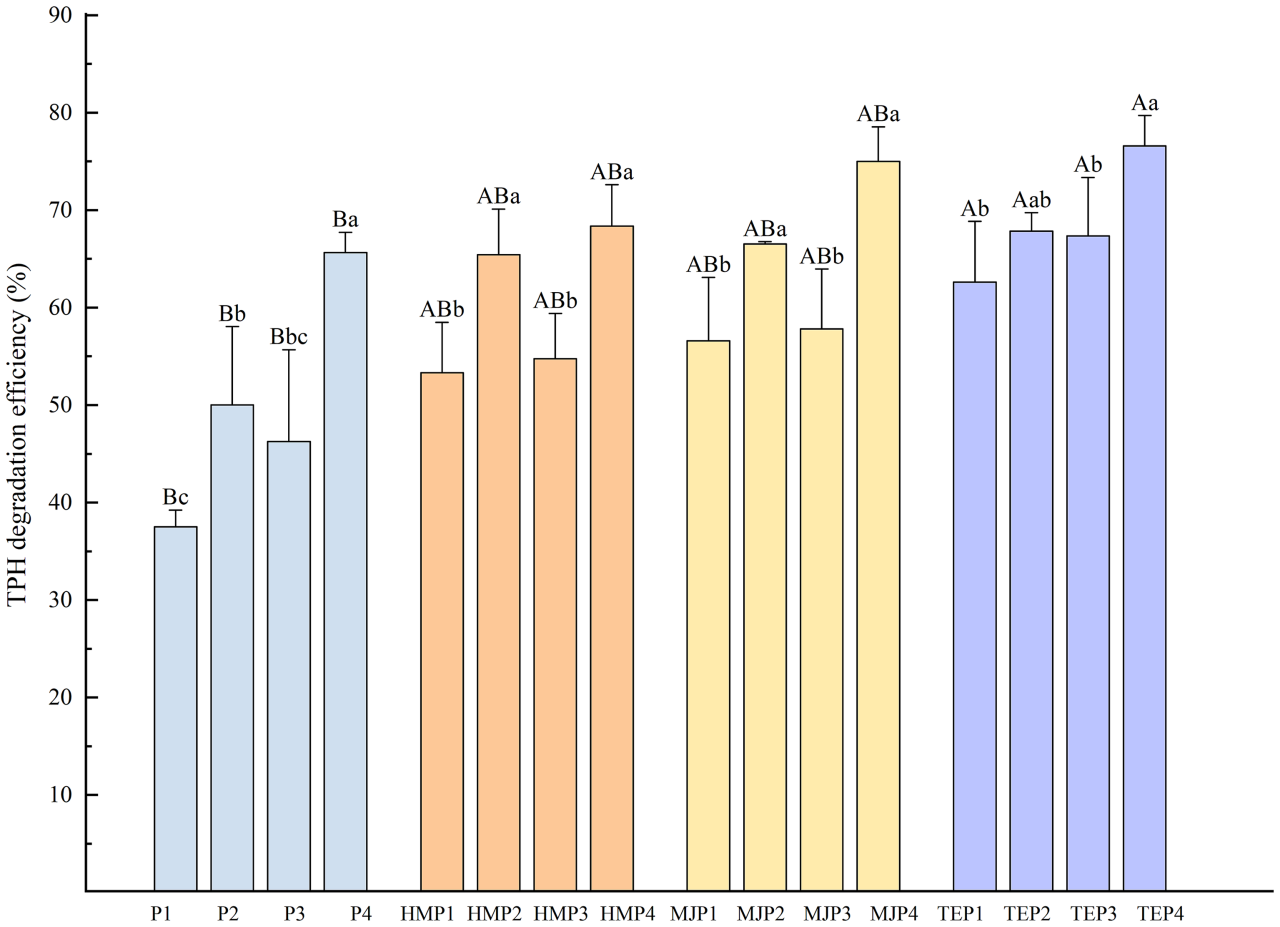
Total petroleum hydrocarbons (TPH) degradation efficiency of soil with different treatments. Different uppercase letters represent the significant differences (*p* < 0.05) among the treatments with different plants or without plants and different lowercase letters represent the significant differences (*p* < 0.05) in the treatments with the same plant but combined with different measures.

Soil total petroleum hydrocarbons (TPH) degradation efficiency after 90 days of treatment with different remediation methods is shown in Figure 1. The treatment without any remediation measures (P1) had the lowest TPH degradation efficiency of 37.53%, which could only degrade petroleum by natural degradation and decomposition by indigenous microorganisms. The addition of microbial agent, biochar, and the planting of plants all significantly increased TPH degradation efficiency (*p* < 0.05). In the treatments with plants, most of the plants showed a better oil degradation effect in co-application with microbial agent than in co-application with biochar. Overall, *T. erecta* was the best plant species in terms of the ability to degrade oil pollution. The highest TPH degradation was achieved by the combined application of *T. erecta*, microbial agent, and biochar, with a value of 76.60%.

To further clarify the interaction effects of plant, microbial agent, and biochar additions on TPH degradation efficiency, a multivariate variance analysis was conducted as shown in Supplementary Table S1. The analysis revealed that plants, microbial agent, and biochar all played an extremely significant role in the degradation of TPH in soil, with the addition of microbial agent being the most influential measure. In addition, the interaction effect between the microbial agent and biochar also significantly affected TPH degradation.

### Soil stoichiometric characteristics

The soil total nitrogen content (TN), total carbon content (TC) and carbon to nitrogen ratio (C/N) after 90 d of restoration in each treatment are shown in Supplementary Table S2. The effect of adding microbial agent and biochar on soil TC and TN was more significant compared with planting plants. The oil-contaminated soil with no remediation treatment (P1), which had the lowest TPH degradation efficiency, had the lowest soil TC and TN contents. In contrast, TC and TN were significantly increased in most of the treatments with biochar addition. The oil-contaminated treatments with no plant had a higher C/N than the treatments planted with any plant, while the treatments planted with *T. erecta*, which had the highest rate of TPH degradation, had the lowest C/N.

### Plant growth

**Figure 2:**
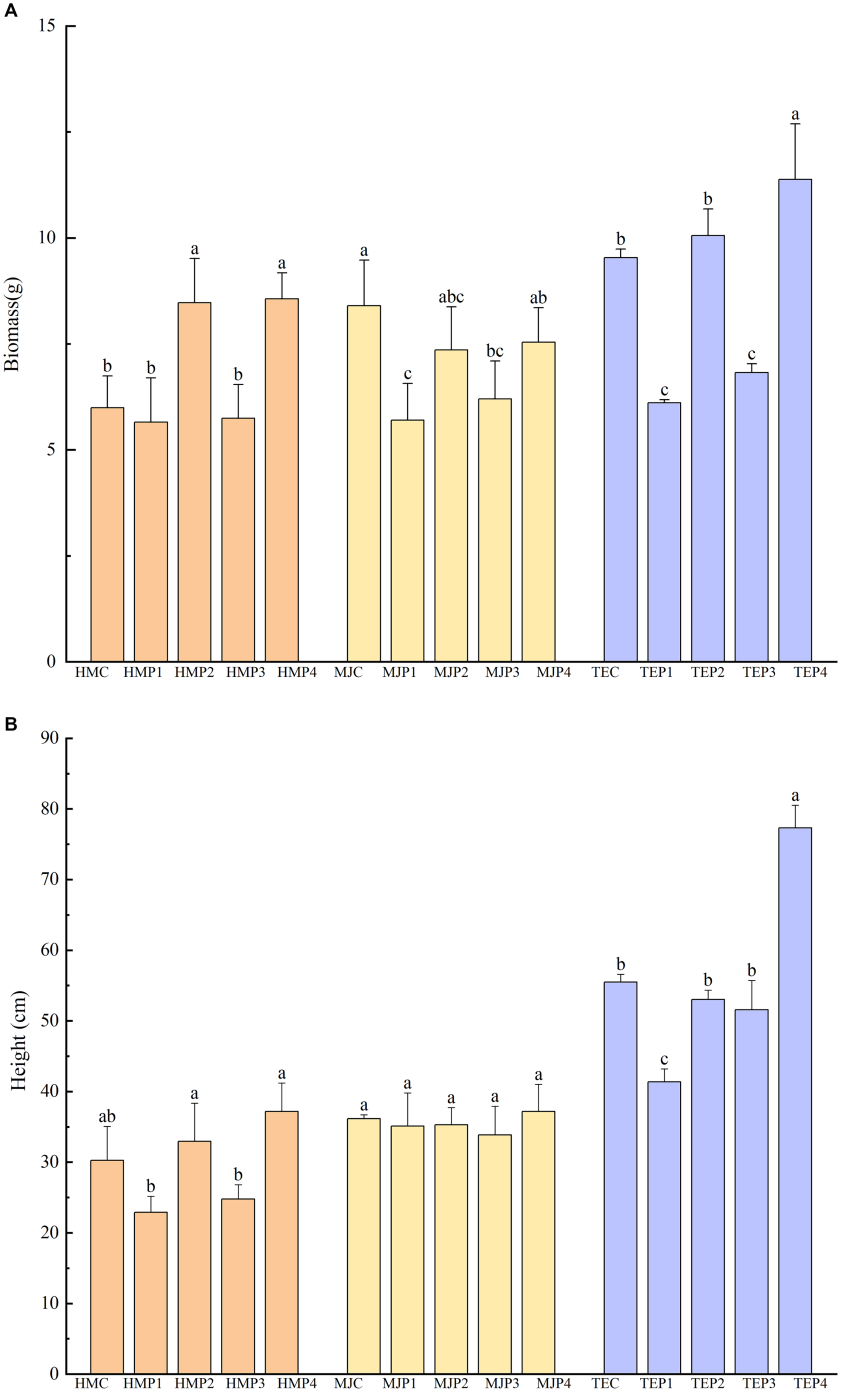
Plant biomass and height of different treatments. Different lowercase letters represent the significant differences (*p* < 0.05) in the treatments with the same plant but combined with different measures. **(A)** shows the plant biomass of different treatments and **(B)** shows the height.

Plants were harvested after 90 d of remediation of oil-contaminated soil by different methods and plant biomass and plant height were determined as shown in Figure 2 and Supplementary Figure S1. For control treatments, plants were grown in the same conditions but without oil-contaminated soil. Biomass and height of the plants in the oil-contaminated treatments were lower or significantly lower than those of the control. However, there was a significant rebound in plant biomass and plant height under combined restoration, especially with the addition of microbial agent or both microbial agent and biochar. In the combined biochar–microbial agent–phytoremediation treatment of *H. moscheutos* (HMP4) and *T. erecta* (TEP4), biomass was elevated to 151.50 and 186.25%, respectively, compared to the phytoremediation alone treatment (HMP1 and TEP1), and plant height was elevated to 201.93 and 184.26%, respectively. However, the addition of biochar alone had little effect on the recovery of plant biomass and height.

### Bacterial community diversity of soils

**Figure 3:**
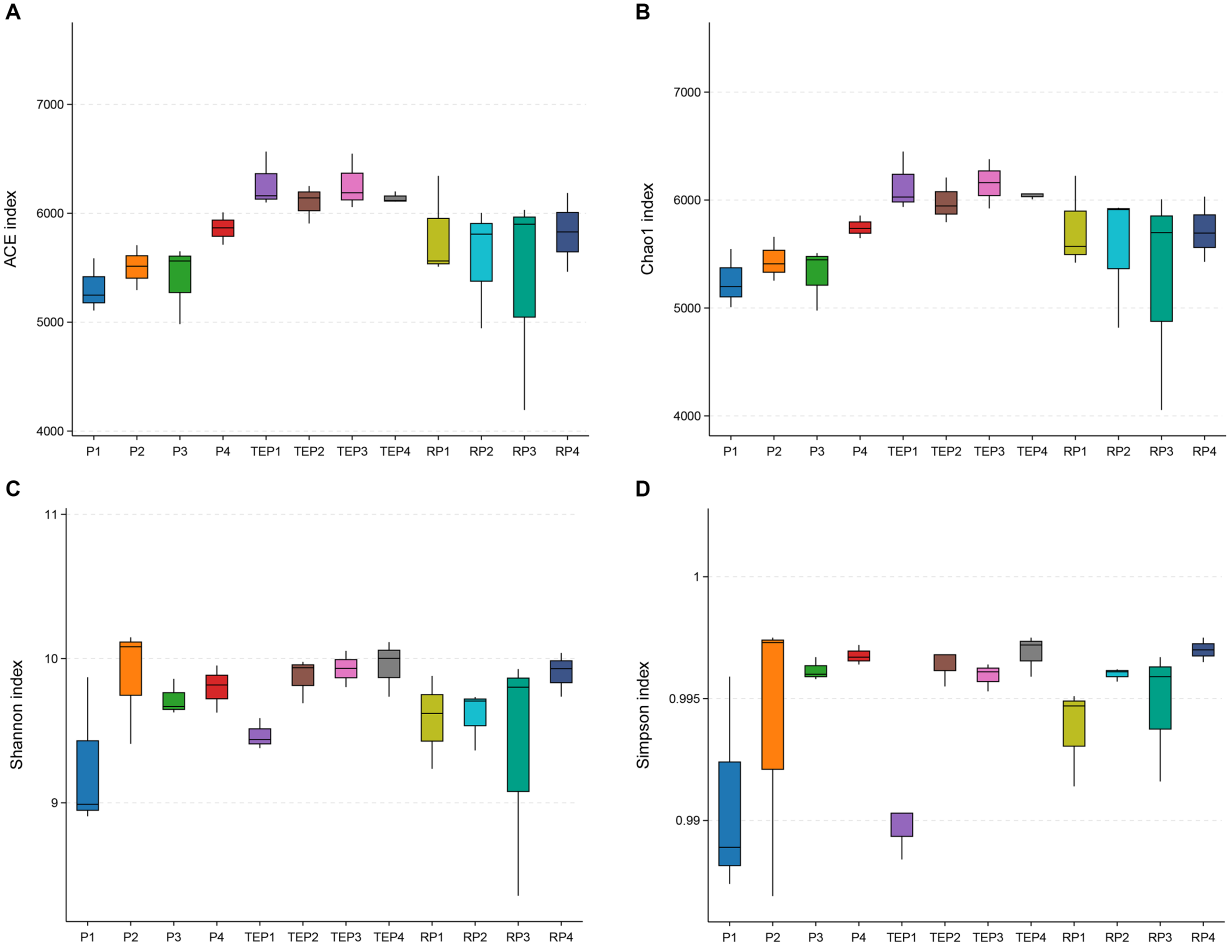
Alpha diversity indexes of the soil microbial community: **(A)**, ACE index; **(B)**, Chao1 index; **(C)**, Shannon index; **(D)**, Simpson index. Horizontal coordinates represent the different experimental treatments. P1, P2, P3, and P4 represent the soils of the treatments without remediation, remediated with microbial agent only, remediated with biochar only, and remediated with microbial agent and biochar, respectively. TEP1, TEP2, TEP3, and TEP4 represent the non-rhizosphere soils of the treatments remediated with *T. erecta* only, remediated with *T. erecta* and microbial agent, remediated with *T. erecta* and biochar, and remediated with *T. erecta*, microbial agent, and biochar combined, respectively. RP1, RP2, RP3, and RP4 represent the rhizosphere soils of the TEP1, TEP2, TEP3, and TEP4 treatments, respectively (the same below). The vertical coordinate is the value of the corresponding alpha diversity index.

*T. erecta*, which exhibited the highest TPH degradation efficiency, was selected to collect samples of rhizosphere and non-rhizosphere soils for comparing with soils grown without plants and analyzing the differences in soil microbial communities. Four diversity indexes in Figure 3 showed the difference in alpha diversity of soil bacterial communities, including the community richness indexes ACE and Chao1, and the community diversity indexes Simpson and Shannon. The higher the indexes, the higher richness and diversity of a community. It was shown that the ACE and Chao1 indexes were lowest in the oil-contaminated soil without any restoration measures (P1), whereas the ACE and Chao1 indexes were higher in the *T. erecta* planted treatment especially in non-rhizosphere soil (TEP1–TEP4) compared with other treatments. For the Simpson and Shannon indexes, P1 was likewise very low. On the contrary, the soil with microbial agent (P2) and the non-rhizosphere soil remediated with *T. erecta*, microbial agent, and biochar (TEP4) had the highest Simpson and Shannon indexes.

**Figure 4:**
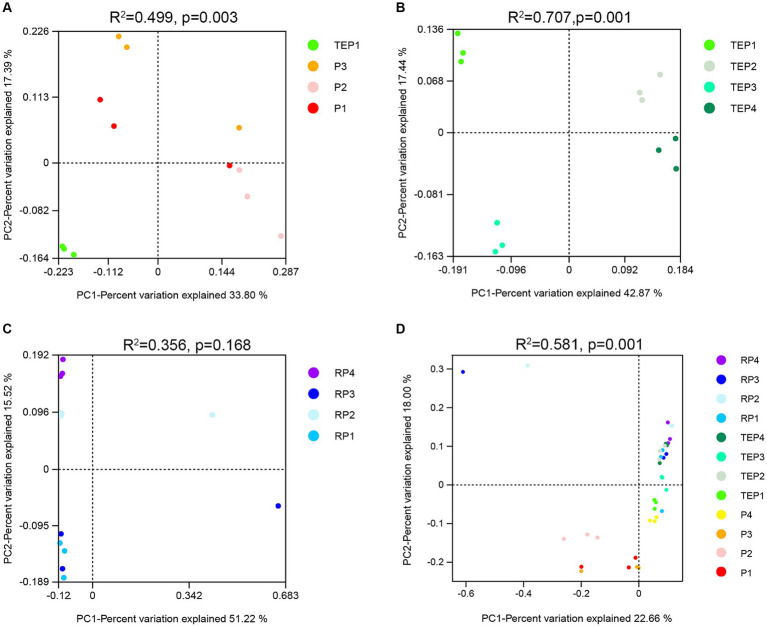
Principal coordinate analysis (PCoA) of bacterial community structures. Each point in the figure represents a sample and different colors represent different treatments. The horizontal coordinate indicates the first principal component, while the percentage indicates the contribution of the first principal component to the sample variance. The vertical coordinate indicates the second principal component, while the percentage indicates the contribution of the second principal component to the sample variance. **(A)** shows the principal coordinate analysis (PCoA) of bacterial communities between each single treatment and the unremediated oil-contaminated soil. **(B)** shows the PCoA of bacterial communities in the nonrhizosphere soils remediated by the combination of plants and other treatments. **(C)** shows the PCoA of bacterial communities in the rhizosphere soils remediated by the combination of plants and other treatment. **(D)** shows the PCoA of bacterial communities in all the soil samples.

To explore the difference in beta diversity of soil bacterial communities, principal coordinate analysis (PCoA) based on Bray–Curtis distance at the OTU level was performed (Figure 4). Significant differences in bacterial communities between the phytoremediation treatment (TEP1) and the other treatments (P1, P2, and P3) were detected (Figure 4A). There were also extremely significant differences in bacterial communities in the non-rhizosphere soils remediated by the combination of plants and the respective treatments (Figure 4B). In addition, the horizontal axis (PC1) separated samples with and without microbial agent, and the vertical axis (PC2) separated samples with or without biochar. Figure 4C indicates that bacterial communities in the rhizosphere (RP1) were not influenced by the addition of biochar (RP3), but were clearly influenced by the addition of microbial agents, either alone (RP2) or in combination with biochar (RP4), as evidenced by the separation of samples along the vertical axis (PC2). As shown in Figure 4D, the separated treatments were P1, P2, and P3, which were not restored or were restored with a single restoration method and none of which had phytoremediation involved.

### Taxonomic analysis of soil bacterial community composition

**Figure 5:**
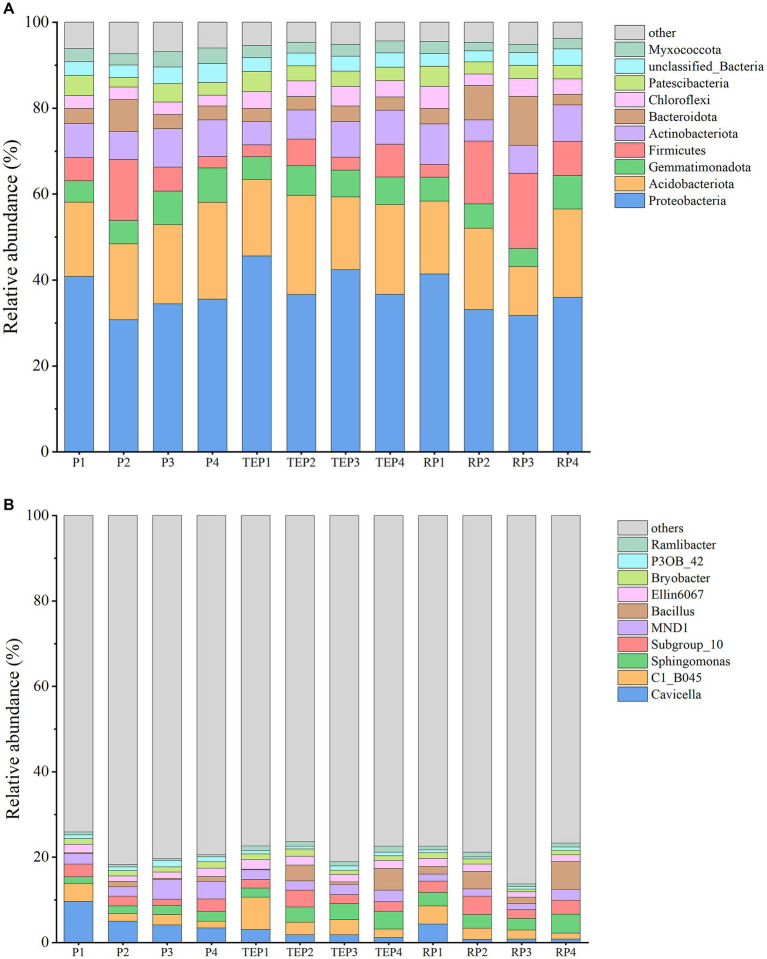
Relative abundance of soil bacteria. **(A)** shows the relative abundance of soil bacteria at phylum level and **(B)** shows the relative abundance of soil bacteria at genus level. Horizontal coordinates are experimental treatments and vertical coordinates are relative abundance percentages. Different colors indicate different phylum or genus and stacked columns are the top10 taxa in relative abundance at each taxonomic level.

At the phylum level, the top 10 dominant taxa were Proteobacteria, Acidobacteriota, Gemmatimonadota, Firmicutes, Actinobacteriota, Bacteroidota, Chloroflexi, Patescibacteria, unclassified_Bacteria, and Myxococcota (Figure 5A). The abundance of the Firmicutes and Bacteroidota was significantly increased in the soil remediated with the addition of microbial agent and in the rhizosphere soil.

At the genus level, the top 10 classified dominant taxa in relative abundance were *Cavicella*, *C1_B045*, *Sphingomonas*, *Subgroup_10*, *MND1*, *Bacillus*, *Ellin6067*, *Bryobacter*, *P3OB_42*, and *Ramlibacter* (Figure 5B). Most of the restored treatments, regardless the methods of remediation (including P2–RP4), showed a decrease in the relative abundance of *Cavicella* and an increase in the relative abundance of *Sphingomonas* compared with the control treatment (P1). Most of the bacteria in the microbial agent used in this study were *Bacillus* and *Micrococcus*. *Micrococcus* was detected in low abundance in this study. However, the relative abundance of *Bacillus* significantly increased in the treatments with microbial agent, especially in the treatments where the microbial agent was applied in combination with plants.

### Correlation analysis between relative abundance of soil bacteria and the effect of soil remediation

**Figure 6:**
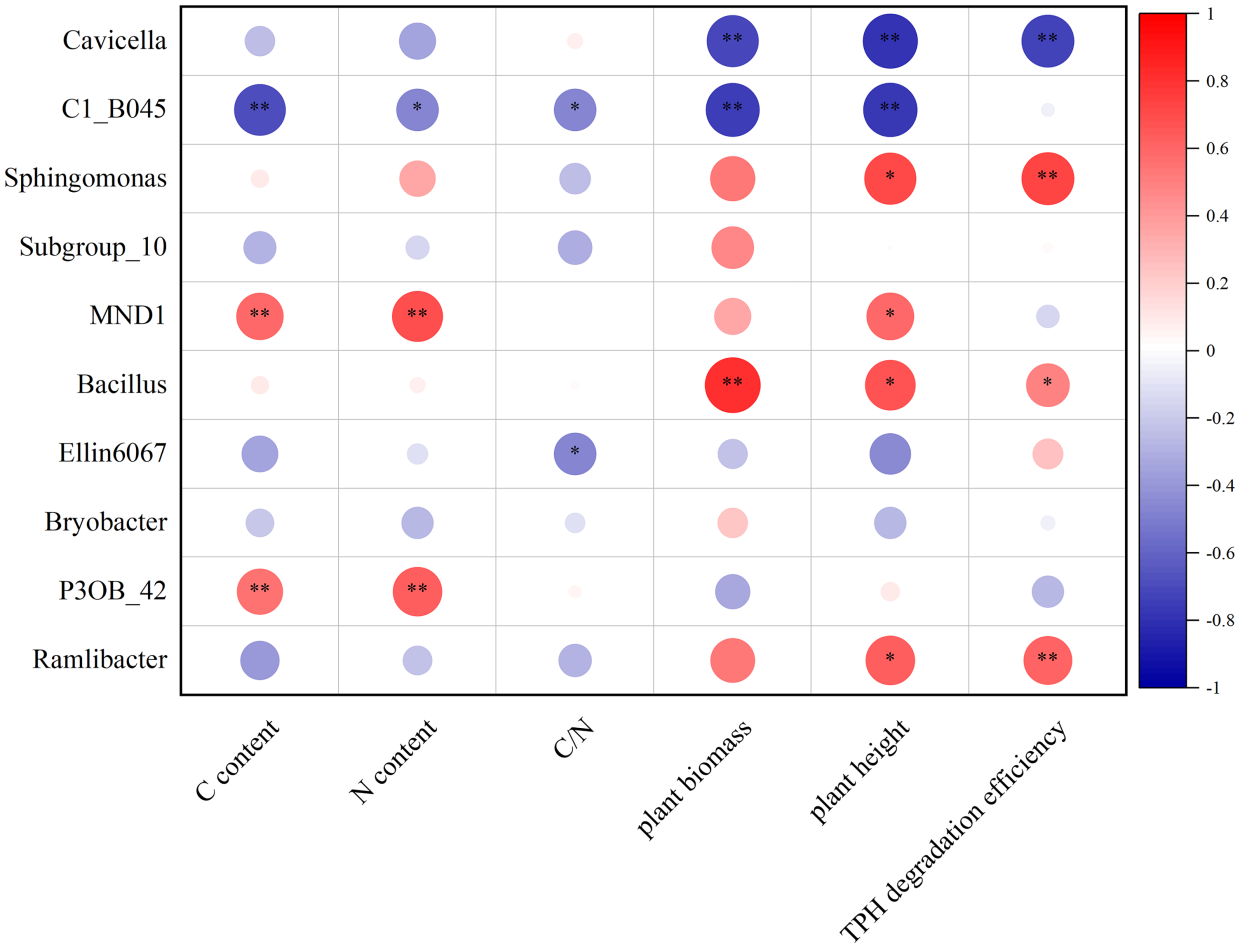
Correlation analysis between relative abundance of soil bacteria and the effect of soil remediation. The vertical coordinate represents the top 10 classified dominant genus in relative abundance at the genus level and the horizontal coordinates are the indicators related to the soil remediation effects detected in this study. Red color indicates positive and blue color indicates negative correlations. *p* level is given: *p < 0.05, **p < 0.01.

The correlation analysis of the top 10 soil bacteria in relative abundance at the genus level with the previously measured indicators related to soil remediation effectiveness is shown in Figure 6. Among these 10 bacteria, *Bacillus* was the only genus that was positively correlated with all soil remediation indicators tested and showed significant or extremely significant positive correlations with TPH degradation efficiency, plant height, and plant biomass. *Sphingomonas* and *Ramlibacter* also showed extremely significant positive correlations with TPH degradation efficiency and significant positive correlations with plant height. For soil physicochemical properties, *MND1* and *P3OB_42* demonstrated extremely significant positive correlations with soil TC and TN contents.

### Difference analysis of soil bacterial community

**Figure 7:**
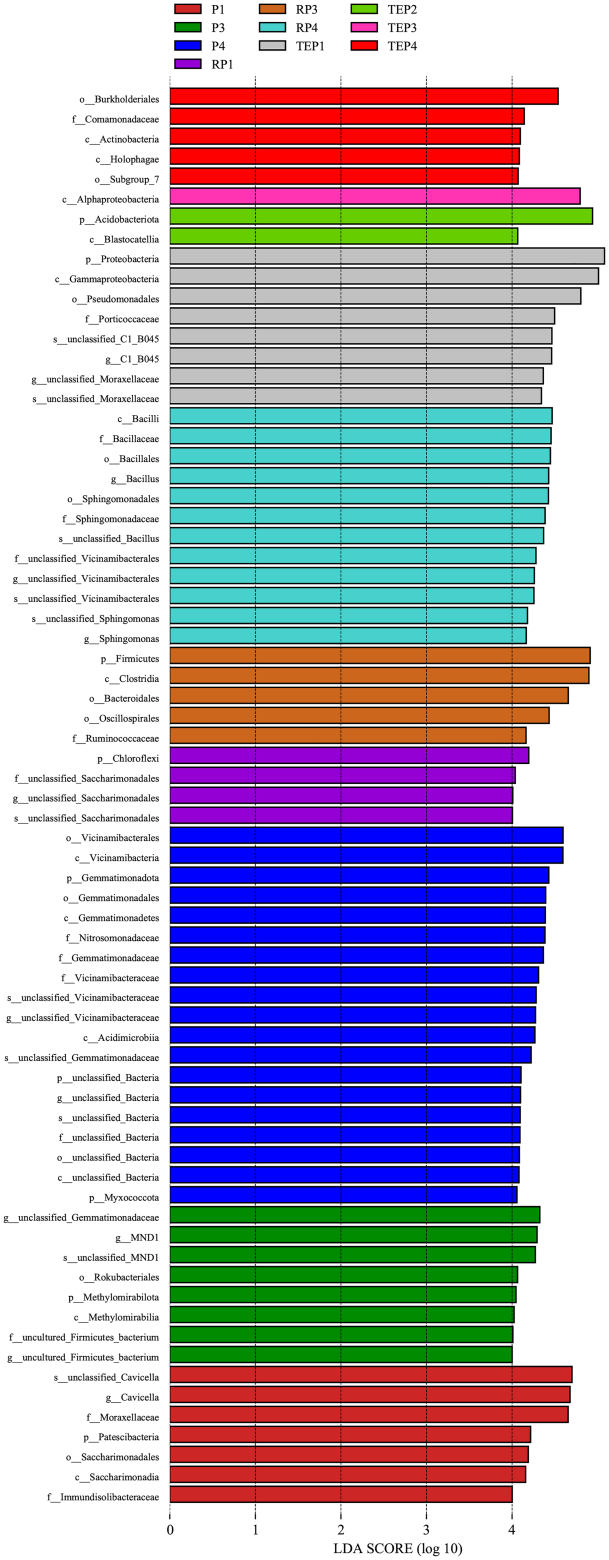
The linear discriminant analysis (LDA) value distribution. The vertical coordinates are the taxonomic units with significant differences between treatments, and the horizontal coordinates visualize the logarithmic scores of the LDA analysis for each taxonomic unit. The taxonomic units are sorted according to the size of their scores, with longer lengths indicating more significant differences of the taxonomic unit, and the color of the bars indicating the treatment corresponding to the higher abundance of that taxonomic unit.

The linear discriminant analysis (LDA) effect size (LEfSe) method was used to determine the taxa most likely to explain differences among the soil bacterial community in different treatments. A total of 71 taxa were identified with significant differences (Figure 7). The oil-contaminated non-rhizosphere soils planted only with *T. erecta* (TEP1) differed from other treatments in being enriched with a high abundance of Proteobacteria, and the rhizosphere soil (RP1) had increased levels of Chloroflexi. Soils from the oil-contaminated treatment with biochar alone (P3) differed from the other treatments due to the presence of *MND1* and Methylomirabilia. The biomarkers for the combined biochar–phytoremediation treatment were Alphaproteobacteria in non-rhizosphere soils (TEP3) and Firmicutes in the rhizosphere soils (RP3). For the combined remediation treatment with biochar, microbial agent, and *T. erecta*, the biomarkers that constituted the difference between that treatment and the other soils were predominantly Burkholderiales and Holophagae in non-rhizosphere soils (TEP4) as well as *Bacillus* and Sphingomonadaceae in rhizosphere soils (RP4).

### Functional gene prediction

**Figure 8:**
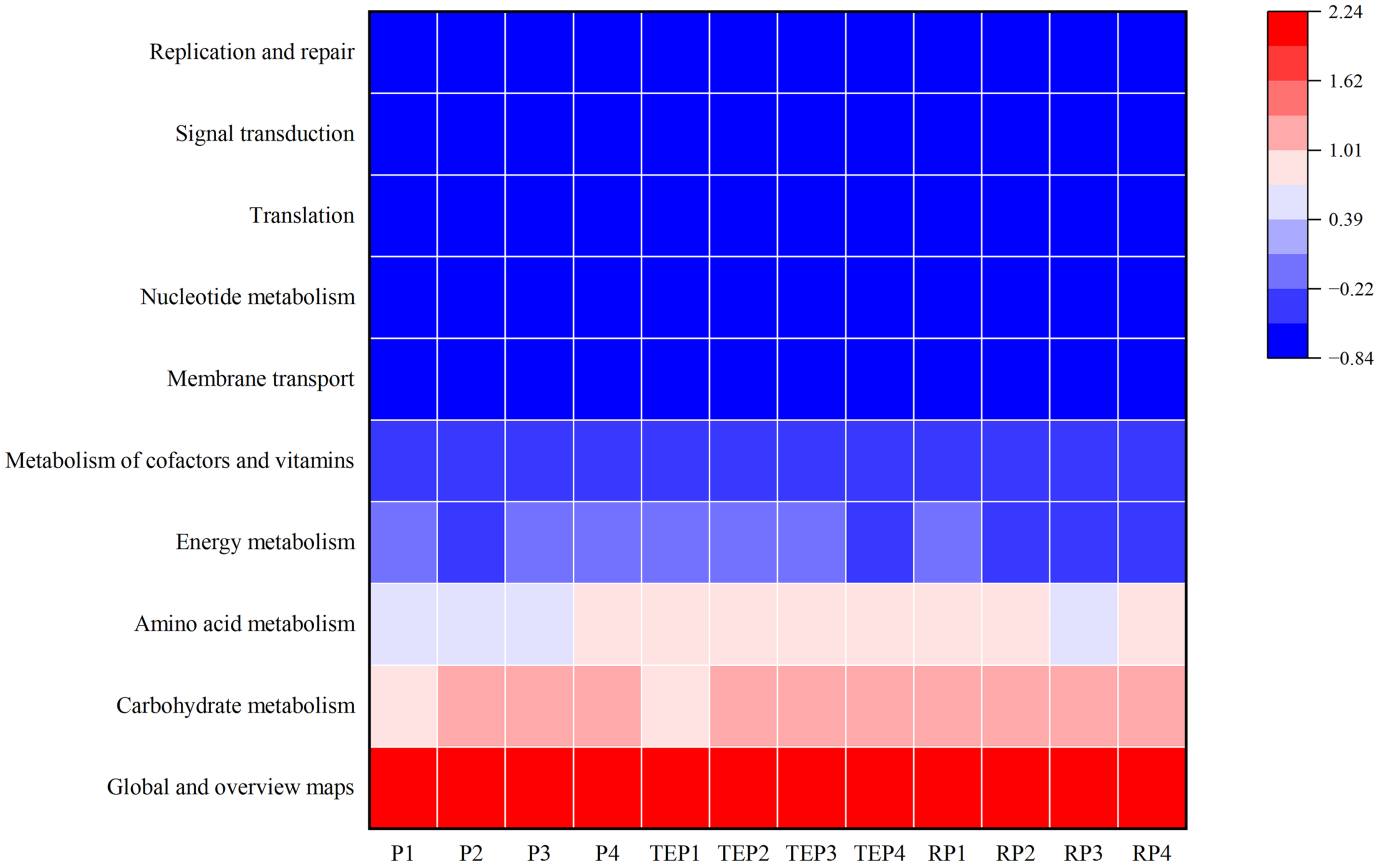
Prediction of functional genes in soil bacteria based on KEGG pathway. Horizontal coordinates represent experimental treatments and vertical coordinates represent KEGG metabolic pathways. Blue to red indicates increasing abundance.

Based on the KEGG database, the secondary functions of soil bacteria were predicted and the top 10 major functional pathways in terms of relative abundance were obtained (Figure 8), with the highest percentage being for global and overview maps. In addition, genes of the carbohydrate metabolism pathway were up-regulated in most treatments except for the oil-contaminated soil without any remediation (P1) or with *T. erecta* remediation alone (TEP1). Genes of the amino acid metabolism pathway were down-regulated in the treatments without phytoremediation (P1, P2, and P3) as well as in the non-rhizosphere soil of the treatment co-remediated by *T. erecta* and biochar (TEP3). In contrast to the performance of the other pathways, genes of the energy metabolism pathway were down-regulated in the soils of the microbial agent remediation treatment (P2) and the combined biochar–bacteria–*T. erecta* treatment (TEP4), which had high efficiency of oil degradation, and were generally in low abundance in plant rhizosphere soils (RP2, RP3, and RP4).

## Discussion

Oil pollution can seriously affect the physical and chemical properties of soil and plant growth. Bioremediation is a valuable approach for tackling oil pollution, but the effect of single bioremediation often fails to meet expectations owing to environmental factors and other issues like actual application methods and complexity of contaminants. In this study, multiple measures were applied in combination, and the most effective remediation method was the combination of biochar, microbial agent, and *T. erecta*, which resulted in an oil degradation rate of 76.60%. *T. erecta* performed better than the other two plants in our study, possibly due to its larger biomass and better recovery of biomass and plant height with the addition of microbial agent or biochar. Plants used for phytoremediation typically have the characteristics of high biomass and resistance to stress (Doroshenko et al., 2019). Ikeura et al. (2016) found that the roots of *Zinnia profusion*, which belongs to the same family (Compositae) as *T. erecta*, could serve as a nutritional source for soil microorganisms in oil-contaminated soils. In this study, *T. erecta* also exhibited better interaction effects with the microbial agent than other plants. Microorganisms are frequently believed to be the main players in co-bioremediation (Xu et al., 2018; Shirzadian Gilan et al., 2023). Our multivariate variance analysis for oil degradation efficiency agrees with this view that the degreasing microbial agents have the highest contribution to the degradation of oil. Our multivariate variance analysis also demonstrated that in terms of the interaction effect, the combined effect of microbial agent and biochar contributed more to oil degradation in this experiment. This is consistent with the findings of other researchers that the combined application of bioaugmentation and biochar is superior to the combined application of biostimulation or phytoremediation with biochar (Dike et al., 2022). This was corroborated by the fact that the increase in TPH degradation efficiency and the recovery of plant growth under combined plant and biochar remediation in this experiment were not significant.

The various remediation techniques all contribute to degrading the pollution and simultaneously restoring the soil physicochemical properties. In this study, the effects of the three remediation measures on the improvement of soil nutrient content were, in descending order: biochar > microbial agent > plants. One possible explanation for this outcome is that the microbial agent itself contains a certain amount of nutrients and the biochar contains a large amount of carbon. On the other hand, biochar is also able to improve the nutrient status of the soil by sequestering carbon and increasing soil porosity and water-holding capacity (Galitskaya et al., 2016; Singh et al., 2022). Our study was conducted under oil-contaminated conditions and crude oil also contains a large amount of carbon, thus, it should not be generalized that a higher carbon content in the soil is preferable (Akachukwu et al., 2018; Mukome et al., 2020). The treatments planted with *T. erecta*—the plant with the highest rate of TPH degradation in this study—did not differ significantly from the other treatments in TC and TN contents, but had the lowest C/N ratio, which may be attributed to maintaining soil nutrients while simultaneously degrading most of the oil.

Soil microbial communities, as sensitive indicators under stress, can be severely affected by oil contamination (Li Q. et al., 2022). The results of our study indicate that planting plants, adding microbial agents, and incorporating biochar all contributed to varying degrees of enhancement in soil microbial richness and diversity compared to oil-contaminated soil without any remediation methods. Wang et al. (2014) found that soil microbial abundance and diversity increased after 12 weeks of combined remediation of soil contamination by bacteria and plants, which is consistent with our study. Among the three measures tested in this study, planting was the most effective measure to increase soil microbial richness, and the combination of all three remediation measures was most effective for increasing the diversity of the soil microbial community. Increases in soil microbial community richness and diversity are conducive to the enhancement of plant resistance to oil pollution and the remediation of oil pollution (Li Q. et al., 2021; Li J. et al., 2021; Li S. et al., 2021). However, the changes in soil microbial communities also highlight the potential unforeseen ecological impacts of introducing exotic microorganisms, and this important issue must be considered in practical applications.

High-throughput sequencing is a valuable tool for investigating the structure and function of soil microbial communities and facilitates understanding of the biological processes involved in the degradation of petroleum hydrocarbons in soil (Wang Q. L. et al., 2020; Gao et al., 2022). In this study, the soil bacterial community structure changed significantly between oil-contaminated and remediated soils. At the phylum level, the most abundant taxon in most of the soil samples was Proteobacteria. Sutton et al. (2013) similarly showed that Proteobacteria was the dominant phylum in oil-contaminated soils. In addition, we found that the following phyla were relatively abundant: Acidobacteriota, which may be related to soil organic matter content (Shelyakin et al., 2022); Gemmatimonadota, which participates in the detoxification and degradation of organic pollutants and has a high tolerance to organic pollutants (Zhang et al., 2022); and Actinobacteriota, which has been reported as one of the most common bacteria in oil-contaminated soils (Egorova et al., 2023).

At the genus level, *Cavicella* with the high relative abundance in this study, is a recently discovered genus that is more likely to be enriched in crude oil-contaminated soils or diesel-contaminated soils and may contain novel petroleum hydrocarbon-degrading bacteria (Xue et al., 2020; Assil et al., 2021). *C1_B045* is still poorly documented although it has been detected in some oil pollution studies (Teske et al., 2022). Peng et al. (2020) found that the highest abundance of *C1_B045* in oil-polluted seawater was accompanied by the highest oxidation rate of polycyclic aromatic hydrocarbons (PAHs). In this study, *Cavicella* and *C1_B045* had the highest relative abundance at the genus level, but the correlation analysis showed negative correlation between their abundance at day 90 of growth and the remediation effects of oil-contaminated soil. However, because oil degradation in soil is not a static process, it cannot be generalized that the data collected at the end of the experiment fully accounts for the taxa involved in degradation. *Cavicella* and *C1_B045* were likely to have made an important contribution toward degradation in the treatments with high degradation rates, but as the soil starts to recover, they were replaced by other taxa, and this could be the reason for the negative correlation. *Sphingomonas* and *Ramlibacter* are well-known petroleum-degrading and plant-beneficial bacteria, respectively, which is consistent with their positive correlation with TPH degradation and plant height in this study (Shen et al., 2016; Li P. F. et al., 2022). In addition, petroleum contamination may nourish *MND1* in soil, but soil type and concentration of contaminated petroleum both affect the abundance of *MND1* (Shen et al., 2018).

*Bacillus* belonging to the phylum Firmicutes is noteworthy in this study. Firmicutes is often considered as one of the most important phyla in the biodegradation of petroleum-contaminated soil and has a degrading effect on petroleum alkanes, some aromatic hydrocarbons, and other stubborn components (Gao et al., 2022; Hou et al., 2022). Most of the strains of degreasing bacteria in the microbial agent used in our study belong to the genus *Bacillus*, and there was a corresponding increase in the relative abundance of *Bacillus* at the genus level in the treatments where the microbial agent was applied, especially in the treatments where it was applied in combination with plants, or with plants and biochar. This likely represented a favorable colonization of the oil-degrading bacteria in the microbial agent, especially if plants and biochar assist in their growth. Yu et al. (2019) also found that Firmicutes responded positively to nutrient amendments in oil contamination remediation, which may explain the higher abundance of *Bacillus* in the co-remediation treatment in this study. Correlation analysis showed that *Bacillus* was positively correlated with all soil remediation indicators tested in our study. In addition, LefSe analysis revealed that *Bacillus* was the dominant genus that constituted the difference in the structure of the bacterial community between the rhizosphere soil of *T. erecta* and other soils, which may indicate that *Bacillus* with the ability to degrade petroleum contamination congregated in large numbers in the rhizosphere of *T. erecta*. Other studies have also found that *Bacillus* aggregated more readily at the rhizosphere and could enhance the metabolic activity of the plant rhizosphere (Dhote et al., 2017; Hashmat et al., 2019).

In addition to the structure of the community, metabolic function is crucial in studies about microbial communities. Prediction of functional genes of soil bacteria based on KEGG data can better reveal the response of bacterial communities to environmental changes and explore possible degradation strategies of bacteria under oil pollution (Wang et al., 2016; Wang Y. G. et al., 2020). The main pathways that differed among treatments in this study were the carbohydrate metabolism pathway and the amino acid metabolism pathway, where gene abundance was positively correlated with the effect of oil degradation, whereas energy metabolism was negatively correlated. The carbohydrate metabolism pathway and the amino acid metabolism pathway are closely related to the maintenance of cellular functions and participate in defense strategies (Lou et al., 2019). Amino acid transport plays a crucial role in defense against stress, and amino acids are also key factors between plant root growth and microbial colonization (Meo, 2013). In addition, genes of the carbohydrate metabolism pathway may be instrumental in plant–microbe interactions. Bhattacharyya and Jha (2012) found that rhizosphere bacteria can promote plant growth by promoting carbohydrate metabolism in plants. Energy metabolism can also help plants and microorganisms to adapt to environmental pressures under oil stress (Xu et al., 2017). Changes in metabolic pathway abundance reflect adjustments in metabolic pathways made by microorganisms in response to environmental stresses to some extent (Zokaei et al., 2021). In this study, the metabolic pathways were adjusted more in favor of the carbohydrate metabolism pathway and the amino acid metabolism pathway in the case of multiple repair measures combined for remediation of oil pollution. However, the abundance of amino acid metabolism pathway genes was relatively low in the rhizosphere soil of the co-remediation treatment of plants and biochar. Thus, the effectiveness of combined plant and biochar remediation requires further exploration.

## Conclusion

In this study, we compared the degradation effects of microbial agent, biochar, and three different plant species and their combined application on soil oil pollution. We selected *T. erecta* as the most appropriate plant to combine with the microbial agent owing to better plant growth and higher efficiency of TPH degradation compared with the other two plants. Addition of biochar enhanced the remediation effect of combined plant–microbe remediation and the remediation technique that combined biochar, microbial agent, and *T. erecta* performed best. High-throughput sequencing was applied to explore the mechanism underlying the interaction between the remediation components and it was found that microbial community diversity and richness were enhanced in the restored soil. Proteobacteria, Acidobacteriota, Gemmatimonadota, Firmicutes, and Actinobacteriota were the phyla with the highest relative abundance found in oil pollution remediation. *Cavicella*, *C1_B045*, *Sphingomonas*, *MND1*, *Bacillus*, *Ramlibacter*, and other genera found in high relative abundance were all associated with petroleum degradation and plant growth promotion. The abundance of *Bacillus* contained in the microbial agent was also significantly increased in the bacterial community of the soil with the addition of the microbial agent and was positively correlated with all soil remediation indicators tested in this study. Furthermore, *Bacillus* was heavily enriched in the rhizosphere of *T. erecta*. Prediction of functional genes in soil bacteria based on KEGG data revealed that adjustments to metabolic pathways made by soil bacteria under the stress of oil contamination were more in favor of the carbohydrate metabolism pathway and the amino acid metabolism pathway. This study provides a potential method for efficient remediation of oil-contaminated soils, and explores the mechanism of the combined effects of plants and microorganisms in degrading soil oil contamination from the perspective of soil bacterial communities.

## Data availability statement

The data presented in the study are deposited in the NCBI under the BioProject PRJNA1106199.

## Author contributions

XF: Conceptualization, Data curation, Investigation, Methodology, Writing – original draft, Writing – review & editing. MZ: Conceptualization, Data curation, Methodology, Writing – review & editing. PZ: Data curation, Investigation, Writing – original draft, Writing – review & editing. HW: Data curation, Investigation, Writing – review & editing. KW: Data curation, Methodology, Writing – review & editing. JL: Methodology, Writing – review & editing. FS: Conceptualization, Data curation, Methodology, Writing – original draft, Writing – review & editing.
