# Supplementary material for: Biochar-bacteria-plant combined potential for remediation of oil-contaminated soil
Source: Front Microbiol. 2024 May 21;15:1343366. doi: 10.3389/fmicb.2024.1343366 (PMC11148334; doi:10.3389/fmicb.2024.1343366)
Supplement: Supplementary file 1 [file Data_Sheet_1.pdf]

# *Supplementary Material*

**Supplementary Table 1.** Multivariate variance analysis of factors affecting the TPH degradation in soil (F-value).

| | F value |
|---|---|
| Plant | 18.671** |
| Microbial Agent | 67.562** |
| Biochar | 18.321** |
| Plant × Microbial Agent | 1.452 |
| Plant × Biochar | 1.542 |
| Microbial Agent × Biochar | 4.162* |
| Plant × Microbial Agent × Biochar | 0.615 |

p-level is given: *p< 0.05, **p< 0.01.

**Supplementary Table 2.** Soil carbon and nitrogen contents.

| | TN (%) | TC (%) | C/N |
|---|---|---|---|
| P1 | 0.07±0.00Ab | 1.38±0.09Ac | 18.46±1.39Ac |
| P2 | 0.07±0.00Ab | 2.09±0.07Ab | 29.06±1.71Aa |
| P3 | 0.11±0.00Aa | 2.36±0.11Ab | 21.45±0.57Abc |
| P4 | 0.11±0.00Aa | 2.73±0.12Aa | 24.1±1.25Ab |
| HMP1 | 0.07±0.01Ab | 1.33±0.03Ac | 18.64±1.23ABb |
| HMP2 | 0.09±0.01Ab | 1.63±0.15Abc | 17.72±0.62ABb |
| HMP3 | 0.1±0.01Ab | 2.18±0.16Ab | 22.9±0.53ABa |
| HMP4 | 0.12±0.01Aa | 2.84±0.29Aa | 23.69±1.76ABa |
| MJP1 | 0.08±0.00Ab | 1.27±0.01Ac | 16.89±0.58ABc |
| MJP2 | 0.08±0.01Ab | 1.74±0.14Ab | 20.91±0.32ABb |
| MJP3 | 0.11±0.01Aa | 2.28±0.13Aa | 20.94±1.03ABb |
| MJP4 | 0.1±0.01Aab | 2.25±0.16Aa | 23.98±1.01ABa |
| TEP1 | 0.07±0.01Ab | 1.39±0.05Ab | 19.3±1.37Ba |
| TEP2 | 0.08±0.00Ab | 1.53±0.02Ab | 18.71±0.37Ba |
| TEP3 | 0.11±0.01Aa | 2.06±0.16Aa | 19.9±0.27Ba |
| TEP4 | 0.1±0.00Aa | 2.19±0.11Aa | 21.56±1.57Ba |

Different uppercase letters represent the significant differences (p< 0.05) among the groups with different plants or without plants and different lowercase letters represent the significant differences (p< 0.05) among the groups with the same plant but combined with different measures.

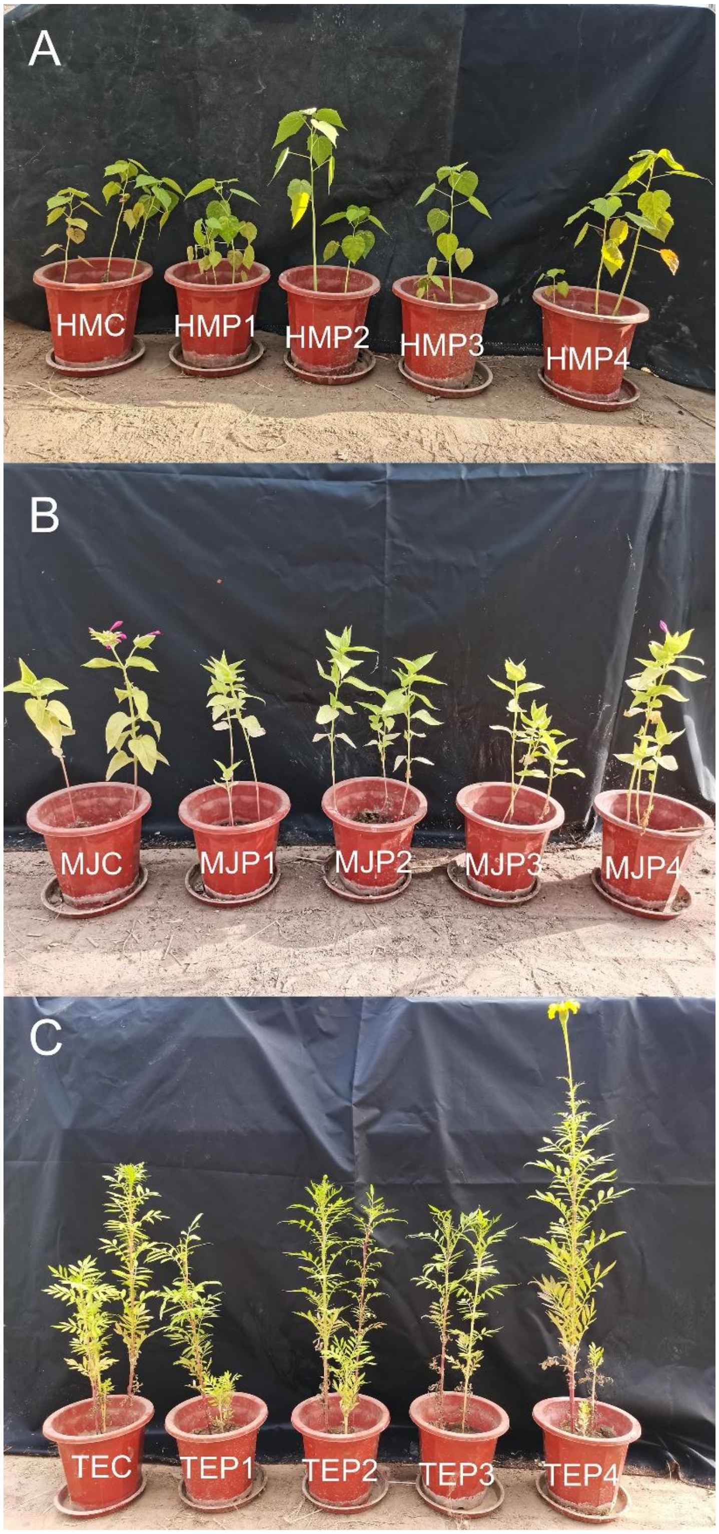


**Supplementary Figure 1.** Growth status of plants.

Figure S1A showed the growth status of *Hibiscus moscheutos*, Figure S1B showed the growth status of *Mirabilis jalapa* and Figure S1C showed the growth status of *Tagetes erecta*. The abbreviations on the pots represent experimental groupings.
